# An Intronic *Alu* Element Attenuates the Transcription of a Long Non-coding RNA in Human Cell Lines

**DOI:** 10.3389/fgene.2020.00928

**Published:** 2020-08-31

**Authors:** Rosario Pérez-Molina, Rodrigo G. Arzate-Mejía, Erandi Ayala-Ortega, Georgina Guerrero, Karin Meier, Fernando Suaste-Olmos, Félix Recillas-Targa

**Affiliations:** Departamento de Genética Molecular, Instituto de Fisiología Celular, Universidad Nacional Autónoma de México, Mexico City, Mexico

**Keywords:** repeat sequences, *Alu* elements, intragenic enhancer, long intergenic non-coding RNAs, transcription attenuation, transposable elements

## Abstract

*Alu* elements are primate-specific repeats and represent the most abundant type of transposable elements (TE) in the human genome. Genome-wide analysis of the enrichment of histone post-translational modifications suggests that human *Alu* sequences could function as transcriptional enhancers; however, no functional experiments have evaluated the role of *Alu* sequences in the control of transcription *in situ*. The present study analyses the regulatory activity of a human *Alu* sequence from the *AluSx* family located in the second intron of the long intergenic non-coding RNA *Linc00441*, found in divergent orientation to the *RB1* gene. We observed that the *Alu* sequence acts as an enhancer element based on reporter gene assays while CRISPR-Cas9 deletions of the *Alu* sequence in K562 cells resulted in a marked transcriptional upregulation of *Linc00441* and a decrease in proliferation. Our results suggest that an intragenic *Alu* sequence with enhancer activity can act as a transcriptional attenuator of its host lincRNA.

## Introduction

Repetitive elements constitute ~50% of the human genome (Lander et al., 2001; Bannert and Kurth, 2004). Different lines of research suggest that they can affect transcriptional regulation, however, most evidence supporting a direct regulatory activity of repetitive elements has been correlative at best (Mallona et al., 2016). The *Alu* subfamily of repetitive elements is a class of primate-specific Short Interspaced Nuclear Elements (SINEs) of ~300 base pair (bp) length that is present in more than 1 million copies in the human genome and hence constitutes the most abundant class of transposable element in humans (Lander et al., 2001). Nevertheless, their role in regulating gene expression and chromatin structure remains poorly characterized.

*Alu* elements are located preferentially in the proximity of gene-rich regions (Batzer and Deininger, 2002; Kim et al., 2016) and are rich in Transcription Factor Binding Sites (TFBS), which suggests a possible function as regulatory platforms for the transcriptional control of host or neighboring genes (Polak and Domany, 2006). In this regard, *Alu* elements have been suggested to nucleate epigenetic silencing *via* the acquisition of DNA methylation and histone post-translational modification H3K9me3, resulting in transcriptional silencing of neighboring genes (Graff et al., 1997; Baylin et al., 1998; Estecio et al., 2012). Recent reports have put forward the idea that *Alu* elements have evolved toward enhancer elements in the human genome. This concerns particularly old *Alu* families like *AluSx*, *AluJo*, and *AluJb*, as they are enriched for the histone post-translational modifications H3K4me1, H3K27ac and have gained transcription factor binding motifs over time (Su et al., 2014). These enhancer-like characteristics are present in a tissue-specific manner and preferentially engage in long-range interactions with gene promoters and with *Alu* sequences thereof. Although these lines of evidence implicate that *Alu* elements, or at least a subset of them, can exert direct regulatory effects in gene transcription, direct characterization of the biochemical regulatory capacity of *Alus* (Chuong et al., 2017) and their function *in situ* need to be explored.

Here, we characterize the regulatory activity of an intronic *Alu* element of the *AluSx* family in the transcriptional gene regulation of the human *Linc000441-RB1* locus in the K562 erythroleukemic cell line. By employing plasmid-based reporter assays of stably transfected pools of cells and single clones coupled with Fluorescence-Activated Cell Sorting (FACS), we show that this *Alu* sequence behaves as an enhancer element protecting against epigenetic silencing for over 100 days of continuous cell culture. Remarkably, CRISPR-Cas9 deletion of the *Alu* element results in strong transcriptional upregulation of the long intergenic non-coding RNA (lincRNA) *Linc000441* with consequences in cell proliferation, suggesting that the *Alu* sequence behaves as a transcriptional *in situ* attenuator. Overall, our results reveal that a single *Alu* sequence can affect gene transcription and cell proliferation. Importantly, biochemical and *in situ* activities could differ and highlight the importance of analyzing both when characterizing repeat sequences. Furthermore, our results underscore the possibility that *Alu* elements have a more widespread role for transcriptional regulation of lincRNAs than previously anticipated.

## Materials and Methods

### Plasmid Constructs

pRB1prom-GFP plasmid contains *RB1* promoter already characterized and cloned into pEGFP plasmid (pEGFP-1; Clontech, Palo Alto, CA; De La Rosa-Velazquez et al., 2007), which is the intergenic region between *Linc00441* and the human *RB1* gene (chr13:48,877,623-48,878,023; h19 version). The closest *Alu* repeat upstream of *RB1* gene promoter (*AluSx1* repeat chr13: 48,874,474-48,874,760) was amplified (Supplementary Table S1) from human lymphocyte genomic DNA and subcloned into the pRB1prom-GFP in two orientations to generate pAlu(5'-3')-RB1prom-GFP and pAlu(3'-5')-RB1prom-GFP plasmids. All the plasmids contain a neomycin-resistance cassette, which allows G418 selection of stably transfected cells. The integrity of all plasmid constructs was verified by DNA sequencing.

### Cell Culture

K562 human erythroleukemic cells were cultured in ISCOVE medium (Invitrogen). K562 cells (K562 ATCC®CCL-243™) were provided by Gary Felsenfeld (National Institutes of Health, Bethesda, Maryland, USA) and were cultured in DMEM. All media contained 10% (v/v) fetal bovine serum (FBS) and 1% penicillin/streptomycin and were maintained in an incubator at 37°C with 5% CO_2_. Human lymphocytes were obtained from peripheral blood of a healthy donor, isolated with Ficoll-Paque Plus 2 (Amersham) following the manufacturer’s instructions. Written informed consent was obtained from this healthy donor.

### Stable Transfection of K562 Cells

K562 cells were washed twice with phosphate buffered saline (PBS) and resuspended in DMEM. A total of 3 × 10^5^ K562 cells were then transferred to a 6-well plate and transfected with 1 μg (1 μg/μl) of corresponding linearized plasmids using Lipofectamine 2000 (Invitrogen, MA, USA) according to the manufacturer’s instructions. After 6 h, 4 ml of non-selective medium was added to transfected cells. Following 48 h of cell recovery, the cells were transferred to media containing 0.9 mg/ml of G-418 (Geneticin, Calbiochem) for selection. Geneticin-resistant pools were analyzed by FACS at different time points (day 0, day 15, day 25, day 40, and day 60) to obtain the percentage (%) of GFP-positive cells and the mean fluorescence intensity from each construct at every time point. Data were analyzed with BD CellQuest Pro software (BD Biosciences). We performed four independent experiments and computed using the Graphpad Prisma Software 7.0. Statistically significant differences in mean fluorescence intensity values between the Alu-containing constructs and the one without it were computed using a two-tailed Mann-Whitney test (*p* < 0.05).

### Cell Clone Isolation

After 48 h of cell recovery, ~5 × 10^5^ (500 μl) of transfected cells were transferred to a cellulose matrix (Methocel, FLuka) containing 0.9 mg/ml of G-418. Individual colonies were picked after 2–3 weeks and expanded in G-418 containing liquid DMEM to perform subsequent experiments. For each construct, we isolated and analyzed 14 independent clones at different points (day 0, day 15, day 30, day 45, day 60, day 80, and day 100) of continuous cell culture for 100 days. The integrity of the transgene was verified by PCR (Supplementary Figure S1C) and Southern blot (data not shown).

### CRISPR/Cas9-Mediated Targeted Deletion

**Figure 1:**
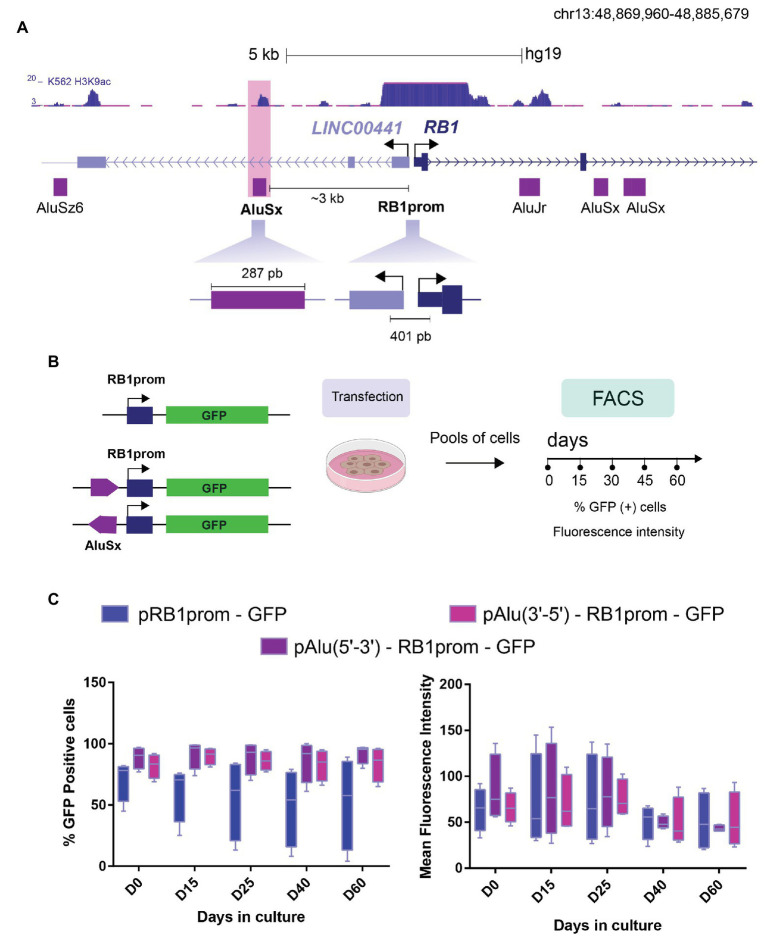
The intrinsic regulatory function of *AluSx* over a promoter. **(A)** Schematic representation of *Linc00441-RB1* locus (chr13: 48,869,960-48,885,679) and localization of *Alu* repeat sequences (purple rectangles). The *AluSx* (highlighted in pink) selected for our analysis is located within the second intron of *LincRNA00441*, about 3 kb upstream of retinoblastoma promoter (*RB1*prom). Both fragments, *AluSx* (283 pb) and *RB1*prom (401 pb) were cloned in GFP reporter plasmid. **(B)** Workflow of the reporter assays showing pRB1prom-GFP and pAlu-RB1prom-GFP plasmid constructs transfected into K562 cells. After selection, we isolated two different cell pools that were analyzed by flow cytometry at different time points of continuous cell culture (Day 0, Day 15, Day 30, Day 45, and Day 60) source icons ©Biorender.com. **(C)** Boxplots that summarize the expression of the GFP reporter gene in K562 cells transfected with different constructs over time. Percentage of fluorescent cells defined by fluorescence-activated cell sorting (FACS) was plotted for each construct for 60 days (left graph). The same cell pools with the corresponding transgenes were evaluated in terms of GFP mean fluorescence intensity (right graph). These graphs represent the data collected from four independent assays (*n* = 4).

*AluSx* element (chr13: 48,874,474-48,874,760, Figure 1A) upstream of the *RB1* gene promoter locus was deleted in K562 cells by co-transfecting two Cas9 containing plasmids plentiCRISPRv2 (Addgene Cat. 52961; Cambridge, MA, USA), each carrying a unique single guide RNA (sgRNA) flanking the *Alu* sequence.

We designed the CRISPR-Cas9 sgRNAs using CRISPOR^1^, as described (Haeussler et al., 2016), to minimize off-target effects. Bsmb1 linkers were added to sgRNAs. The oligonucleotides were then annealed following a standard protocol (Sambrook and Russell, 2006), ligated into the vector and confirmed by sequencing prior transfection. See Supplementary Table S1 for the list of sgRNAs sequences.

The plasmidic vectors were transfected into cells by using Lipofectamine 2000 (Invitrogen) as per manufacturer protocol. Cultures were then selected for 3–4 days with puromycin (5 μg/ml; Sigma). We obtained genomic DNA from CRISPR-Cas9 pools of transfected cells and screened by PCR to confirm the deletion of the evaluated fragment.

Then, CRISPR-Cas9 pools of transfected cells were seeded at low density to isolate monoclonal cell clones with respective mutations through serial dilution in a 96-well plate. Forty-eight randomly selected puromycin-resistant colonies were individually expanded and splitted for future culture and genomic DNA isolation. A hundred nanograms of genomic DNA from these cell clones or K562 genomic DNA (control) were screened by PCR with primer pairs annealing to the region outside of the double-strand break (DSB) sites. See Supplementary Table S1 for the list of screening primers used to confirm targeted deletions. PCR reactions were evaluated on 1% agarose gels. Bands were excised from the gel and purified using the QIAquick Kit (QIAGEN) and the status of the deletion was confirmed by Sanger sequencing. We selected three homozygous clones for two different deletions of the *AluSx* repeat, for further analysis (ΔAlu-C1, ΔAlu-C2, and ΔAlu-C3).

### RNA Extraction and Real-Time Quantitative PCR

Total RNA was extracted from K562 cells with TRIzol Reagent (Invitrogen) according to the manufacturer’s instructions. RNA concentrations were determined using the NanoDrop ND-1000 spectrophotometer (NanoDrop Inc., DE, USA).

Real-time quantitative PCR (RT-qPCR) was carried out with KAPA SYBR® FAST One-Step qRT-PCR kit and specific primers for *RB1*, *Linc00441*, and *α-tubulin* as an endogenous normalization control (Supplementary Table S1). The qPCR reactions were carried out in the StepOne detection system (Applied Biosystems) at 42°C for 5 min, 95°C for 30 s, followed by 40 three-step cycles of 95°C for 3 s, 62°C for 10 s, and 72°C for 10 s, in triplicate for each sample. Relative RNA levels were calculated using the comparative ΔΔCt method (Schmittgen and Livak, 2008). Statistically significant differences in gene expression between the wild-type and the CRISPR mutants were computed using a *t*-test (*p* < 0.05) and the Graphpad Prisma Software 7.0.

### Cell Proliferation Assay

We used Trypan Blue assay to determine cell viability by counting viable cell numbers with a microscope in the following cell lines: K562 WT vs. ΔAlu-C1, ΔAlu-C2, and ΔAlu-C3 (Strober, 2015). On day 0, we started with 1 × 10^5^ cells in a volume of 3 ml by triplicate. We counted in a hemocytometer the cell number for each condition every 24 h during 4 days. Finally, we plotted the average values from triplicates of cell number counts as a function of time for the different cell lines.

### Bioinformatic Analysis

#### Motif Data Analysis

Identification of binding sites for TFs was done using the JASPAR (Fornes et al., 2020) Vertebrate Database using a threshold *p* < 0.0001.

## Results

### An Intronic *Alu* Element Behaves as an Enhancer and Protects Against Epigenetic Silencing in Reporter Constructs

To investigate the regulatory function of *Alu* sequences, we chose the well-characterized *RB1* gene locus. We and others have previously shown, different epigenetic mechanisms are at play to ensure proper control of *RB1* gene expression (De La Rosa-Velazquez et al., 2007; Dávalos-Salas et al., 2011). We hypothesized that the *Alu* sequences closest to the *RB1* gene promoter could impact its transcriptional regulation *via* two general mechanisms. Firstly, induced epigenetic silencing, as it has been reported that young *Alu* elements are epigenetically repressed (ref) and *Alu* sequences can gain DNA methylation in cancer cells (Akers et al., 2014; Bakshi et al., 2016; Jorda et al., 2017) and secondly, transcription boosting by acting as enhancers, as has been proposed for old *Alu* families (Su et al., 2014). Therefore, we retrieved the location of *Alu* sequences surrounding the minimal *RB1* gene promoter including sequences 5 kb upstream and downstream of the transcription start site (TSS), based on the annotation by Repeat Masker (Stirzaker et al., 1997; Figure 1A). The two *Alu* repeats closest to the *RB1* gene promoter are an *AluSx* and an *AluJr* element. The *AluSx* element is located 3 kb upstream of the *RB1* TSS and lies within the second intron of *Linc00441*, a lincRNA divergent to *RB1*, that has been shown to affect *RB1* transcription in cancer cells (Tang et al., 2017). The *AluJr* element is positioned in the first intron of *RB1* gene, about 2.5 kb downstream of the *RB1* TSS. Both *Alu* elements are characterized by multiple TFBS (Supplementary Figure S1) and are enriched for the euchromatin and associated histone post-translational modification H3K9ac. Notably, the H3K9ac, a histone mark associated with active enhancers, was recently found enriched in *Alu* elements expressed in a cell-type specific manner (Zhang et al., 2019). However, only *AluJr* partially overlaps with a region annotated as a promoter in the *RB1* locus based on Chromatin Segmentation by HMM from ENCODE (ENCODE Project Consortium, 2012). Additionally, it is immediately next to a *FLAM* SINE element, posing technical challenges for the manipulation of this sequence (Supplementary Figure S1C). Due to the *AluJr* sequence overlapping with other potential regulatory elements in the *RB1* locus, we dismissed working with the latter and decided to focus our study on the regulatory activity of the *AluSx* element upstream of *RB1* TSS.

Initially, to characterize the regulatory activity of the *AluSx in vitro*, we cloned the *Alu* element in both orientations (5'-3' and 3'-5') in a reporter plasmid containing the *RB1* promoter sequence (RBprom) and *GFP* as a reporter gene (Figure 1B). We have previously employed this reporter plasmid to monitor the epigenetic silencing of the *RB1* gene promoter (De La Rosa-Velazquez et al., 2007). Reporter constructs were transfected into K562 cell line and selected with geneticin to obtain pools of cells with stable integrants that were then evaluated by FACS. As expected, more than 50% of cells transfected with the pRB1prom-GFP construct were GFP-positive [GFP(+); Figure 1C, left, day 0], which is consistent with the promoter activity of this sequence. Unexpectedly, the mean number of GFP(+) cells carrying the *Alu*-containing construct in both orientations (pAlu(5'-3')-RB1prom-GFP and pAlu(3'-5')RB1prom-GFP) was higher than the one observed in pRB1prom-GFP cells (89 and 82%, pAlu-RB1prom vs. 71%, pRB1prom; Figure 1C, left, day 0). A similar trend was observed when analyzing mean fluorescence intensity (86 and 66, pAlu-RB1prom vs. 64, pRB1prom Figure 1C, right, day 0). Importantly, we observed this regulatory effect in four independent experiments, strongly suggesting that the *AluSx* sequence behaves as an enhancer element in this reporter assay. The increase in the number of GFP(+) cells irrespective of the *Alu* sequence orientation is an effect well-characterized for enhancer elements.

We have previously described the progressive epigenetic silencing of stably integrated transgenes over time in cell cultures (Dávalos-Salas et al., 2011). Since the acquisition of epigenetic silencing can be a time-dependent process, it was evaluated if the *Alu* element could still enhance transcription of the reporter gene despite their epigenetic silencing over time. Therefore, we followed pools of cells with stable integrants for the pRB1prom-GFP and pAlu-RB1prom-GFP transgenes over 60 days and quantified the number of GFP(+) cells. As expected for the *RB1* gene promoter, we observed a time-dependent reduction in the mean number of GFP(+) cells (71%, day 0 vs. 52%, day 60) and mean fluorescence intensity (64, day 0 vs. 50, day 60) indicative of epigenetic silencing of the *RB1* gene promoter as we have reported before (Dávalos-Salas et al., 2011). Remarkably, the presence of the *Alu* sequence upstream of the *RB1* gene promoter protected against epigenetic silencing. Accordingly, 92% of the pAlu-RB1prom-GFP cells were GFP(+) on day 60, in sharp contrast to just 52% GFP(+) cells containing the pRB1prom-GFP construct (Figure 1C, left). This effect is less evident at the level of mean fluorescence intensity, which is highly maintained in the pAlu-RB1prom-GFP cells during the first 25 days, and then decreases to the levels of pRB1prom-GFP cells (Figure 1C, right).

Finally, we also evaluated the regulatory activity of the *AluSx* in cell clones with stably integrated constructs and analyzed them for 100 days of continuous cell culture. Consistent with our results in pools of cells, we observed that the *Alu* sequence behaves as an enhancer and protects against epigenetic silencing although showing an increased variability, probably reflecting the effect of integration into different chromatin environments (Supplementary Figure S2). In summary, plasmid-based reporter assays suggest that the *Alu* sequence can act as an enhancer increasing the probability that more cells will become more transcriptionally active, opposed to promoting the number of transcription events in a specific population or acting as a protector against epigenetic silencing (Blackwood and Kadonaga, 1998).

### *In situ* Deletion of the Intronic *Alu* Sequence Results in Changes in Transcription

**Figure 2:**
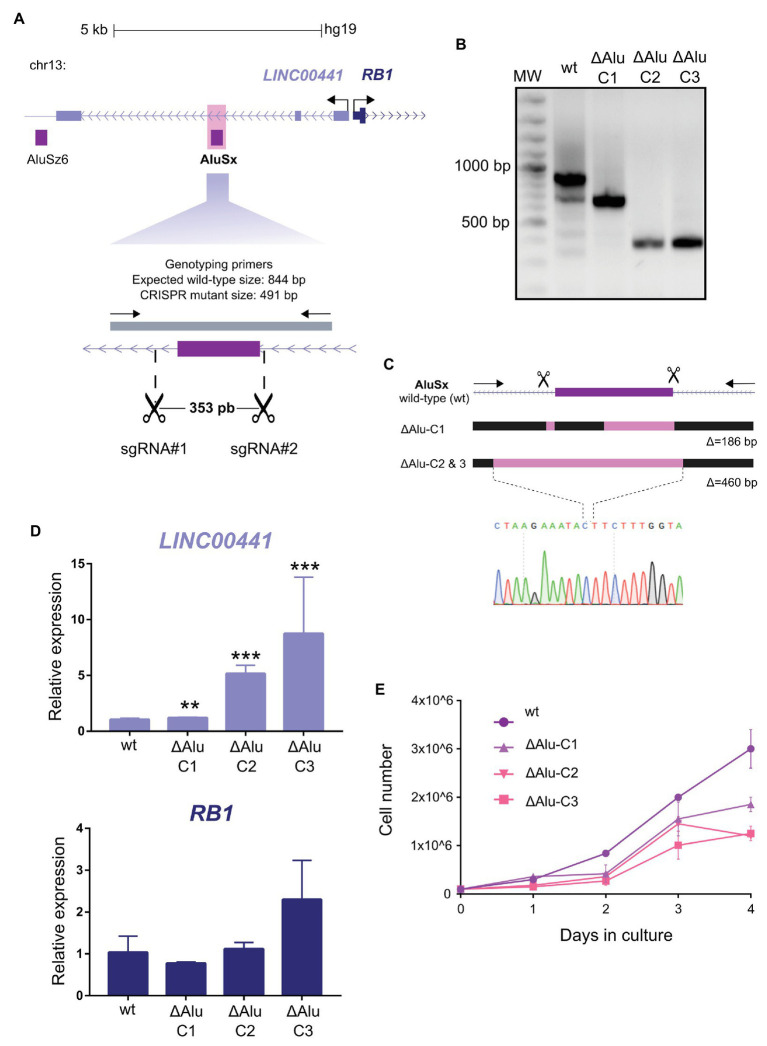
Functional contribution of *AluSx* to the *Linc00441* expression levels. **(A)** Design of CRISPR/Cas9 mediated deletion of *AluSx* in the *Linc00441*-*RB1* locus. The purple rectangle represents the *Alu* repeat. Scissors indicate the target sites of sgRNAs used to generate the deletion. The gray bar depicts the fragment amplified by the genotyping primers (arrows). **(B)** Genotyping of CRISPR mutant clones with deletions spanning the *AluSx* repeat. Expected wild type amplicon size, 844 base pairs (bp). Three CRISPR mutant clones ΔAlu-C1, ΔAlu-C2, and ΔAlu-C3 homozygous for two different deletions of the *Alu* repeat. **(C)** Schematic representation of CRISPR mutant clones ΔAlu-C1, ΔAlu-C2, and ΔAlu-C3 with deletions of the *AluSx* repeat. Pink rectangles represent the deleted sequence in each mutant. The electropherogram of the sequencing at the deletion breakpoints of ΔAlu-C2 and ΔAlu-C3 mutant clones is shown. Δ = the number of base pairs deleted in each CRISPR mutant clone. **(D)** Gene expression analyses of *Linc00441* and *RB1* gene by real-time quantitative PCR (RT-qPCR). Transcriptional quantifications in each CRISPR mutant compared to wild type using *Linc00441* Exon 1 (upper graph) and *RB1* (bottom graph) gene specific primers. Significant differences between wild type and CRISPR mutants were calculated using a *t*-test *n* = 3, ^**^p < 0.01, ^***^p < 0.001. Error bars represent the standard deviation (SD) of three independent experiments at least (*n* = 3). **(E)** Quantification of cell proliferation by a trypan blue cell counting assay. The graph shows the average viable cell numbers of three replicates counted for 4 days on the three CRISPR mutant clones (ΔAlu-C1, ΔAlu-C2, and ΔAlu-C3) compared to wild type.

To assess the *in situ* function of the *AluSx* element, we employed the CRISPR-Cas9 system to generate a deletion of the *Alu* sequence in K562 cells. Using two sgRNAs targeting flanking sequences of the *AluSx* element, we generated a deletion of 353 bp (Figure 2A). After transfection, drug selection, and clonal dilution, we isolated three homozygous clonal cell lines that showed two different molecular lesions (Figures 2B,C). The mutant clone ΔAlu-C1 is characterized by a deletion of 186 bp that removes 172 bp of the 3' region of the *AluSx*. In contrast, the mutant clones ΔAlu-C2 and ΔAlu-C3 have a deletion of 460 bp that removes the *Alu* sequence completely, as well as an additional 150 bp 5' upstream of the repeat (Figure 2C and Supplementary Figure S2). Next, we evaluated the effect of the deletion on the transcription of *RB1* and the host gene *Linc00441* of the deleted *AluSx* sequence. We found that in the three mutant clones the transcription of *RB1* gene is only marginally affected, showing a tendency toward an increase that did not reach statistical significance. Unexpectedly, *Linc00441* expression was strongly upregulated; in particular, the increase was higher in the clones that lack the entire *AluSx* element (Figure 2D). Given our results in reporter constructs that suggest that *AluSx* possesses an intrinsic enhancer activity, the intragenic *AluSx* could be acting as an intragenic enhancer of *Linc00441*, which attenuates the host gene expression. Similar observations have been made for intragenic enhancers of protein-coding genes in humans (Cinghu et al., 2017).

Since *Linc00441* has been involved in cancer (Tang et al., 2017), we evaluated the effect of the *AluSx* element deletion on cell proliferation in mutant clones. The deletion of the *AluSx* element resulted in a decrease of K562 proliferation (Figure 2E). It is worth mentioning that when we analyzed the effect that *Alu* removal had on our three mutant clones, we observed different behaviors between ΔAlu-C1 and ΔAlu-C2/ΔAlu-C3 both on the level of gene expression levels and cell proliferation.

It was proposed that *Alu* elements could also participate in transcription regulation by providing multiple TFBS when inserted in gene-rich regions (Polak and Domany, 2006). Therefore, we carried out an analysis of TFBS on the *AluSx*, identifying the ones that remained intact in clone C1 and not in clones C2 and C3 (Supplementary Figure S3). As we expected, we found several TFBS previously reported to be enriched in *Alu* sequences (Norris et al., 1995; Polak and Domany, 2006; Bouttier et al., 2016). Interestingly, the six TFBS that were absent in clones C2 and C3 was SREBF1, ESR1, Gfi1b, CTCFL, VDR, and TFAP2C that could have an essential role for these *AluSx* functioning in transcriptional regulation of *Linc00441*.

Taken together, the *in situ* deletion of the *AluSx* sequence promotes the transcription of the *Linc00441* gene, but not *RB1* gene, and importantly inhibits cell proliferation. This strongly suggests that *Alu* sequences can impact transcription and thereby change cellular phenotypes, such as cell proliferation.

## Discussion

Recent studies have revealed a link between *Alu* elements and the control of gene expression (Hanke et al., 1995; Mallona et al., 2016; Chen et al., 2018). Many of these findings came from extensive computational analyses of genome-wide epigenomic and transcriptomic data (Goerner-Potvin and Bourque, 2018; Zhang et al., 2019); however, the direct functional testing of *Alu* sequences and their contribution repeat to gene regulatory networks have remained poorly explored.

Here, we provide evidence that an *Alu* repeat can behave as an enhancer and protect against epigenetic silencing using reporter assays. Furthermore, partial deletion or the complete removal of the *AluSx* from its endogenous locus increases the transcription of its host lincRNA, affecting cell proliferation. These findings contribute to our understanding of the regulatory potential these primate-specific sequences have in gene expression on the human genome.

In this study, we focused on the *AluSx* repeat located upstream of the *RB1* gene promoter and within *Linc00441*. The presence of this *Alu* repeat increases both the number of GFP-positive cells and the mean fluorescence intensity, suggesting that it acts as an enhancer element. In support of the aforementioned, a recent study analyzed genome-wide nucleosome occupancy, histone modification, and sequence motif features at *Alu* elements, concluding that *Alu* elements showed enhancer features (Su et al., 2014). Interestingly, the effect of the *AluSx* was more evident in the increase of GFP-positive cells instead of the mean fluorescence intensity. This supports the idea that *AluSx* repeat increases the burst frequency of transcription according to the binary model, where enhancers increase transcriptional levels of associated promoters (Blackwood and Kadonaga, 1998). Of note, while our data using plasmid-based reporters to test the regulatory potential of the *AluSx* suggest an effect in transcription and protection against epigenetic silencing, we cannot discard that the *AluSx* sequence, outside of its genomic context, could act as a non-specific DNA spacer that affect epigenetic silencing of the RB-1 promoter.

Although we demonstrated the *cis*-regulatory effect of the *Alu* in reporter assays, these experiments are naturally limited by the fact that the repeat sequence is investigated independently of its native chromosomal context. Therefore, we chose to determine its role in the regulation of its host and neighboring genes and its implications in cell proliferation, through a combination of CRISPR-Cas9 mediated deletions and gene expression analysis. Contrary to the classical function of an enhancer element, we observed in mutants lacking the *AluSx* sequence a significant increase in the expression of *Linc00441*, but not *RB1*. From this, we conclude that *AluSx* must attenuate its host gene expression as reported for intragenic enhancers of protein-coding genes in humans (Cinghu et al., 2017). Notably, this attenuator effect was observed specifically for intragenic enhancer-containing genes with low-to-moderate expression levels in embryonic stem cells, reasoning that the enhancer’s dominant function is presumably the one of an attenuator at these genes.

A recent report raised another important aspect for enhancer activity of TE that depends on the cooperative action of multiple TFs, whose binding motifs appear to have been already present in the corresponding ancestral TE insertions (Sundaram et al., 2017). We performed an analysis of the motifs of TFBS present in the *AluSx* repeat element. We found that many of the TFBS identified, are binding sites for nuclear factors, hormones ligands as well as other TFs related to differentiation processes, which correlates with motifs that have been reported to be enriched on *Alu* sequences (Polak and Domany, 2006). Interestingly, we found that six TFBS remain intact in the mutant that carry a partial deletion of the *AluSx*, probably related to the modest effect observed on expression and proliferation, compared with the mutants that have a complete removal of *AluSx*. Among these TFBS, we identified binding motifs related with metabolic pathways (SREBF1; sterol biosynthesis), hormone response elements (ESR1 and VDR) and differentiation processes (Gfi1b in hematopoietic lineage and TFAP2C in early morphogenesis). Interestingly, we also identified a TFBS for CTCF paralog (CTCFL) that can be related with the protective effect against epigenetic silencing observed to this *AluSx*. However, how these TFs are involved in the enhancer activity of this *Alu* repeat is currently unknown. Trying to identify differential contribution of each TFBS related to the attenuator activity may help to get a better understanding of this novel function of repeat elements.

Given the recent observations that *Alu* sequences present enrichment of histone post-translational modifications associated with enhancer elements or the binding of RNA Pol II/III in a tissue or cell-type-specific manner (Su et al., 2014; Zhang et al., 2019), it would be of great interest to investigate the role of the *AluSx* in other cell-types. Such an experiment would inform on the presence of specific factors, such as cell-type specific transcription factors that impact the regulatory activity of an *Alu* sequence.

Further studies are required to investigate, on a genome-wide scale, the net impact of intragenic *Alu* in lincRNA expression. Nevertheless, our findings suggest that these repeat elements may be part of the complex machinery fine-tuning transcriptional regulation, highlighting the need for more functional assays to unravel the mechanisms of these enigmatic elements.

## Data Availability Statement

The raw data supporting the conclusions of this article will be made available by the authors, without undue reservation.

## Ethics Statement

Written informed consent was obtained from the donor.

## Author Contributions

RP-M performed the experiments with the support of RA-M, EA-O, GG, and FS-O. RP-M RA-M, and FR-T analyzed the data and performed the statistical analysis. RP-M, RA-M, and FR-T conceived the study, participated in its design, and wrote the manuscript. KM and EA-O critically revised the manuscript. All authors have read and approved the final version of the manuscript.

### Conflict of Interest

The authors declare that the research was conducted in the absence of any commercial or financial relationships that could be construed as a potential conflict of interest.

## Footnotes

^1^http://crispor.tefor.net/

## Supplementary Material

SUPPLEMENTARY FIGURE S1 *Alu* elements surrounding the *Linc00441*-RB1 locus are enriched for TF binding sites and show enrichment for H3K9ac. **(A)** Genomic landscape surrounding the *Linc00441*-*RB1* locus. Data derived from the UCSC Genome Browser. **(B)** Genomic landscape of the *AluSx* element upstream of the *RB1* gene promoter and located in the intron 2 of *Linc00441*. **(C)** Genomic landscape of the *AluJr* element located in the intron 1 of *RB1*.

SUPPLEMENTARY FIGURE S2 The intrinsic regulatory effect of *AluSx* on a promoter. **(A)** Representative flow cytometry profiles of K562 cellular pools expressing the transgenes described in Figure 1B after 0, 15, 30, 45, and 60 days of continuous cell culture. **(B)** Schematic workflow of the isolation of individual cell lines carrying each one of the transgene reporter constructs. Fourteen cell independent clones were isolated after selection and the integrity of transgenes in each cell line was confirmed by PCR and Southern blotting (data not shown). Fourteen independent clones from each GFP-transgene were analyzed by FACS at different time points (Day 0, Day 15, Day 30, Day 45, Day 60, Day 80, and Day 100) of continuous cell culture (Source icons ©Biorender.com). **(C)** Amplification using three different primer pairs (depicted as blue, green, and orange arrows) was performed on genomic DNA obtained from each cell line. The blue and green primers were used to verify the integrity of the transgenes. Single and multi-copy integrants were determined using the orange primers. The expected amplicon lengths are indicated below the arrows. At the bottom a representative gel for the three PCRs is shown. **(D)** Boxplots that summarize the expression of the GFP reporter gene in the 14 isolated cell lines (*n* = 14) obtained for each construct over time. The percentage (%) of fluorescent cells (upper graph) and the mean fluorescence intensity (bottom graph) determined by flow cytometry are shown. Significant differences between the different constructs were calculated using a Mann-Whitney test, with confidence level set as 95%. ^**^*p* < 0.01.

SUPPLEMENTARY FIGURE S3 Analysis of motifs in *AluSx* and their removal in CRISPR mutants. Schematic representation of the region (chr13: 48,874,274-48,874,960) that contains the *AluSx* element upstream of the *RB1* gene promoter. Light purple rectangles represent the deleted region in each mutant (**top panel**). Motif binding sites in *AluSx* (chr13: 48,874,474-48,874,760) by MEME (*p* < 0.0001) are shown as boxes in light purple. Highlighted region in pink corresponds to the non-deleted sequence in the ΔAlu-C1 mutant allele, with a partial deletion of the *AluSx*, containing six TFBS (**middle panel**). Logo motifs from each TFBS with their corresponding *p* are shown (**bottom panel**).
