# Supplementary material for: An Intronic Alu Element Attenuates the Transcription of a Long Non-coding RNA in Human Cell Lines
Source: Front Genet. 2020 Aug 31;11:928. doi: 10.3389/fgene.2020.00928 (PMC7489498; doi:10.3389/fgene.2020.00928)
Supplement: Supplementary file 1 [file Data_Sheet_1.pdf]

NAME/IDENTIFIER	FORWARD PRIMER	REVERSE PRIMER
**Primers used for PCR amplification of RB1-promoter sequence (used in the reporter plasmid assay; related to Figure 1)**		
Fp/Rupp	5’-CGGGATCCAGACTCTTTGTATAGCC-3’	5’-CGGGATCCCGAGCTGTGGAGGAG-3’
**Primers used for PCR amplification of AluSx sequence (used in the reporter plasmid assay; related to Figure 1)**		
Alu(S1)	5’-GATGGGGTCTTGCTCTTG-3’	5’-CTAGACCAGGTACGGTGG-3’
**Guide RNA sequences used for CRISPR/Cas9-mediated targeted deletions (related to Figure 2)**		
5prime_alu	5’-CACCGGTGAATTTCAAGAAGTTCAGG-3’	5'-AAACCCTGAACTTCTTGAAATTCACC-3'
3prime_alu	5’-CACCGGATTTCGGCAGAGTCTAGACC-3’	5'-AAACGGTCTAGACTCTGCCGAAATCC-3'
**Screening primers used to test CRISPR/Cas9-mediated targeted deletions (related to Figure 2)**		
Alu screen big	5'-GGAAGCAGAGAGAACCAATGG-3'	5'-CACACCACCATAGCACACAT-3'
Alu screen small	5'-AGGAGTCTTACAGCAATCTTCTT-3'	5'-CATCCACACAACTCAGAAGCA-3'
**Gene-specific primers used for RT-qPCR analysis (related to Figures 2)**		
RB-RT	5'-GGATCAGATGAAGCAGATGGAAG-'3	5'-GCATTCGTGTTCGAGTAGAAGTC-'3
LincRNA00441-Ex1-RT	5'-GGCTGGGACGCTAAGTCATG-'3	5'-GGGGTGGTTCTGGGTAGAAG-3'
aTUB-RT	5'-TGGAACCCACAGTCATTGATGA-'3	5'-TGATCTCCTTGCCAATGGTGTA-'3
