# Supplementary figures and images for: An Intronic Alu Element Attenuates the Transcription of a Long Non-coding RNA in Human Cell Lines
Source: Front Genet. 2020 Aug 31;11:928. doi: 10.3389/fgene.2020.00928 (PMC7489498; doi:10.3389/fgene.2020.00928)

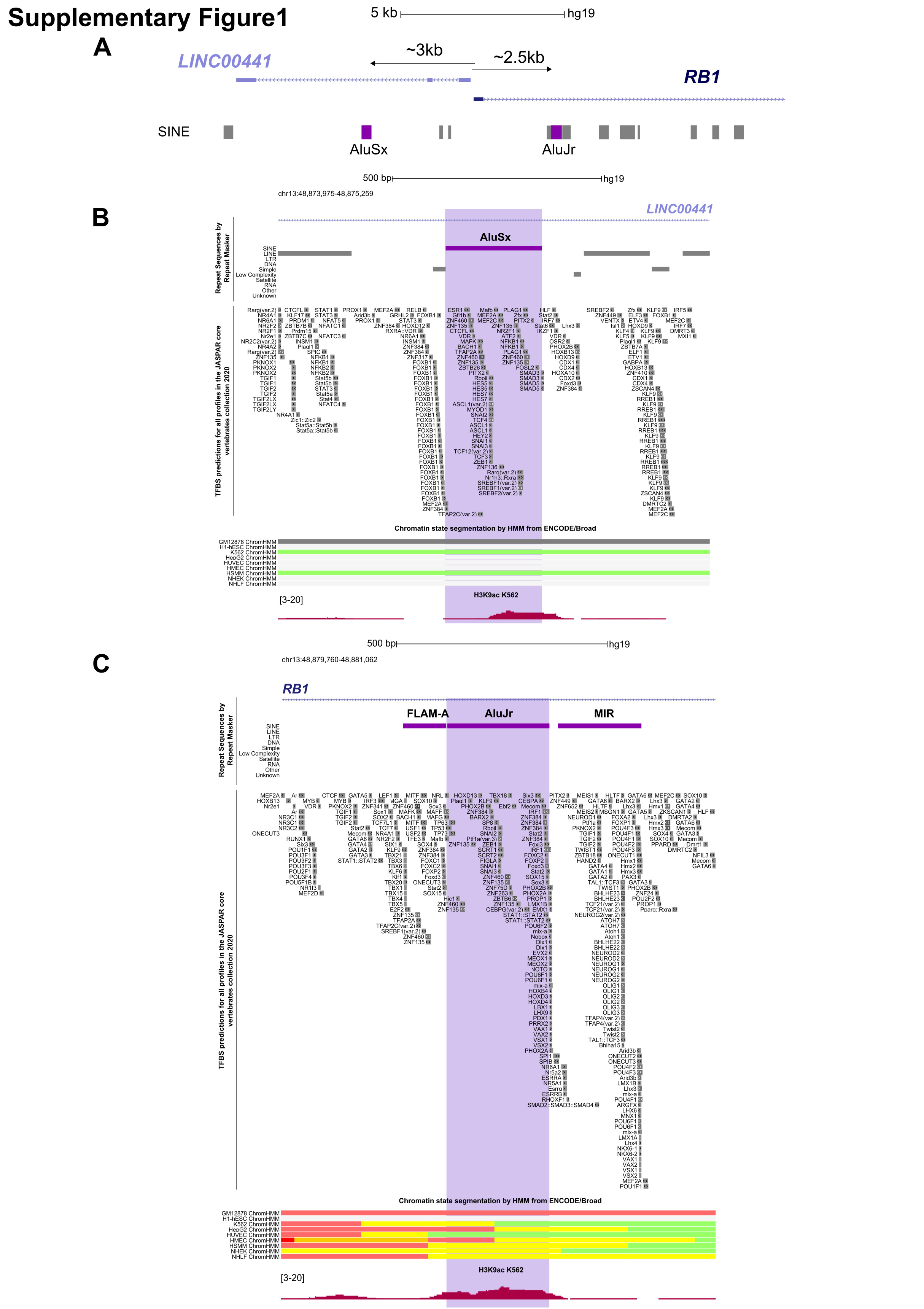

Supplement: SUPPLEMENTARY FIGURE S1 — Alu elements surrounding the Linc00441-RB1 locus are enriched for TF binding sites and show enrichment for H3K9ac. (A) Genomic landscape surrounding the Linc00441-RB1 locus. Data derived from the UCSC Genome Browser. (B) Genomic landscape of the AluSx element upstream of the RB1 gene promoter and located in the intron 2 of Linc00441. (C) Genomic landscape of the AluJr element located in the intron 1 of RB1. [file Image_1.JPEG]

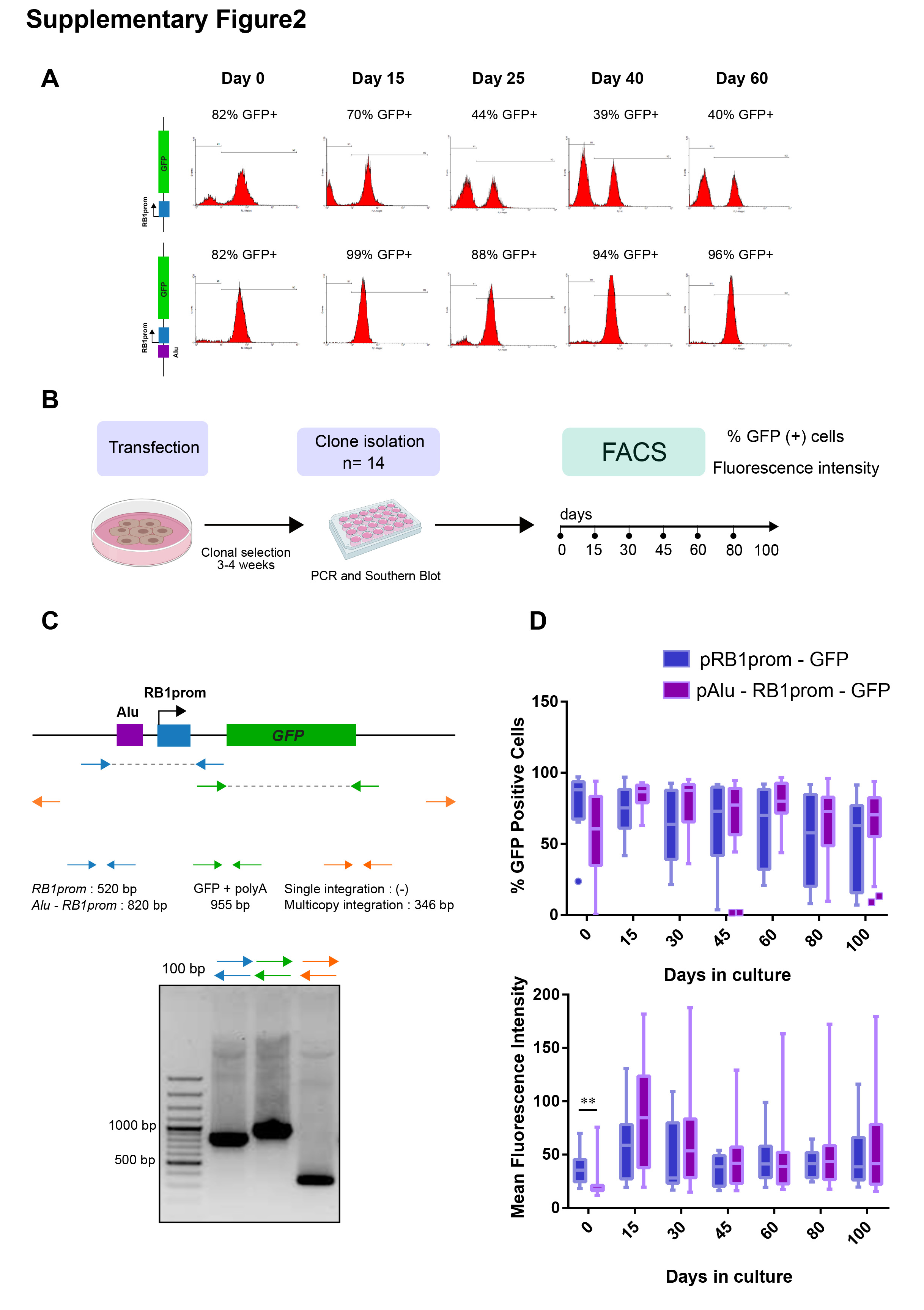

Supplement: SUPPLEMENTARY FIGURE S2 — The intrinsic regulatory effect of AluSx on a promoter. (A) Representative flow cytometry profiles of K562 cellular pools expressing the transgenes described in Figure 1B after 0, 15, 30, 45, and 60 days of continuous cell culture. (B) Schematic workflow of the isolation of individual cell lines carrying each one of the transgene reporter constructs. Fourteen cell independent clones were isolated after selection and the integrity of transgenes in each cell line was confirmed by PCR and Southern blotting (data not shown). Fourteen independent clones from each GFP-transgene were analyzed by FACS at different time points (Day 0, Day 15, Day 30, Day 45, Day 60, Day 80, and Day 100) of continuous cell culture (Source icons ©Biorender.com). (C) Amplification using three different primer pairs (depicted as blue, green, and orange arrows) was performed on genomic DNA obtained from each cell line. The blue and green primers were used to verify the integrity of the transgenes. Single and multi-copy integrants were determined using the orange primers. The expected amplicon lengths are indicated below the arrows. At the bottom a representative gel for the three PCRs is shown. (D) Boxplots that summarize the expression of the GFP reporter gene in the 14 isolated cell lines (n = 14) obtained for each construct over time. The percentage (%) of fluorescent cells (upper graph) and the mean fluorescence intensity (bottom graph) determined by flow cytometry are shown. Significant differences between the different constructs were calculated using a Mann-Whitney test, with confidence level set as 95%. **p < 0.01. [file Image_2.jpg]

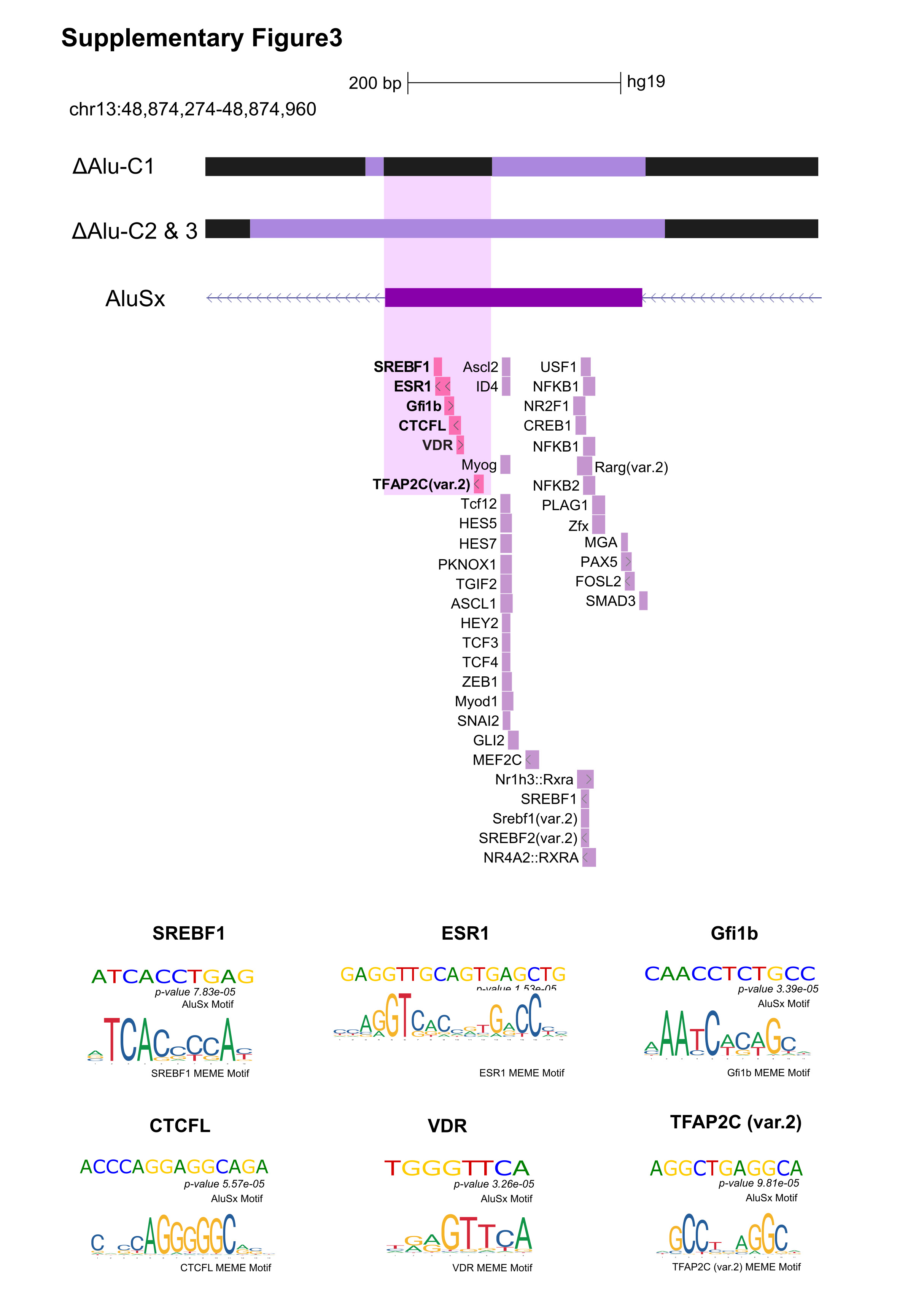

Supplement: SUPPLEMENTARY FIGURE S3 — Analysis of motifs in AluSx and their removal in CRISPR mutants. Schematic representation of the region (chr13: 48,874,274-48,874,960) that contains the AluSx element upstream of the RB1 gene promoter. Light purple rectangles represent the deleted region in each mutant (top panel). Motif binding sites in AluSx (chr13: 48,874,474-48,874,760) by MEME (p < 0.0001) are shown as boxes in light purple. Highlighted region in pink corresponds to the non-deleted sequence in the ΔAlu-C1 mutant allele, with a partial deletion of the AluSx, containing six TFBS (middle panel). Logo motifs from each TFBS with their corresponding p are shown (bottom panel). [file Image_3.JPEG]
